# Spike substitutions E484D, P812R and Q954H mediate ACE2-independent entry of SARS-CoV-2 across different cell lines

**DOI:** 10.1371/journal.pone.0326419

**Published:** 2025-08-01

**Authors:** Greta Vizgirda, Alexander P. Underwood, Ulrik Fahnøe, Nina Weis, Santseharay Ramirez, Jens Bukh

**Affiliations:** 1 Copenhagen Hepatitis C Program (CO-HEP), Department of Infectious Diseases, Copenhagen University Hospital, Hvidovre, Denmark; 2 Department of Immunology and Microbiology, Faculty of Health and Medical Sciences, University of Copenhagen, Copenhagen Hepatitis C Program (CO-HEP), Copenhagen, Denmark; 3 Department of Infectious Diseases, Copenhagen University Hospital, Hvidovre, Denmark; 4 Department of Clinical Medicine, Faculty of Health and Medical Sciences, University of Copenhagen, Copenhagen, Denmark; Telethon Institute of Genetics and Medicine, ITALY

## Abstract

Severe acute respiratory syndrome coronavirus 2 (SARS-CoV-2), which causes coronavirus disease 2019 (COVID-19), has evolved into variants with multiple spike protein coding mutations that affect its transmissibility, infectivity, and immune evasion, in particular from neutralizing antibodies. Several of these amino acid changes have been associated with reduced dependency on the principal angiotensin converting enzyme-2 (ACE2) receptor for cell entry. The present study investigates the role of spike protein changes observed in a cell-culture adapted SARS-CoV-2 isolate (DK-AHH1) in modulating entry, ACE2 dependency, and neutralization across different cells, including human liver and lung cell lines. Using a pseudoparticle system, spike proteins with substitutions E484D, P812R, Q954H, and deletion Δ68−76 were evaluated in Vero E6 and Huh7.5, as well as in A549 cells with and without ACE2 overexpression. Pseudoparticles carrying E484D or P812R individually permitted entry in Huh7.5 cells, and their combination further enhanced this capacity. ACE2 blocking experiments revealed the differential roles of these mutations in mediating entry across cell lines. In Vero E6 cells, P812R was the primary driver for ACE2-independent entry, while E484D facilitated ACE2-independent entry in Huh7.5 cells. In A549 cells, all three substitutions (E484D+P812R + Q954H) were required for ACE2-independent entry. Addition of the Δ68−76 deletion did not increase infectivity in any cell line. Notably, pseudoparticles carrying these mutations, maintained susceptibility to neutralization by convalescent plasma from subjects with COVID-19, regardless of the cell line used. These findings highlight the adaptability of SARS-CoV-2 in utilizing alternative entry mechanisms across various cell types, with E484D and P812R playing critical roles in ACE2-independent entry in cell culture. Overall, this study provides valuable insights into how SARS-CoV-2 can alter its receptor usage to ensure robust infectivity of human cell lines while preserving neutralization sensitivity, contributing to our understanding of viral evolution, and informing potential therapeutic strategies targeting viral entry.

## Introduction

Severe acute respiratory syndrome coronavirus 2 (SARS-CoV-2) and its associated disease, termed coronavirus disease 2019 (COVID-19), has claimed the lives of millions worldwide [1]. Since the outbreak began, SARS-CoV-2 has evolved into multiple variants, each with differing transmissibility, virulence, and resistance to immune responses [2]. These variants are largely distinguished by differences in the surface-located spike protein, which plays a critical role in viral entry and is the primary target for neutralizing antibodies [3]. The spike protein consists of two subunits, S1 and S2. The S1 subunit contains the receptor binding domain (RBD) and is responsible for binding to host cell receptors, while the S2 subunit contains a furin cleavage site that facilitates fusion between the viral and host membranes [4]. SARS-CoV-2 primarily enters target cells by binding to angiotensin converting enzyme-2 (ACE2), which is ubiquitously expressed throughout the human body [5]. Entry occurs either via a cathepsin-mediated manner or via direct fusion to the host cell membrane with the presence of both ACE2 and transmembrane serine protease 2 (TMPRSS2) [6].

Although SARS-CoV-2 is considered a respiratory pathogen, its tropism is not limited to respiratory tissues. Both *in vivo* and *in vitro*, SARS-CoV-2 has been shown to infect numerous cell types, including podocytes, hepatocytes, and enterocytes [6–8]. The most common cell type used for *in vitro* studies of SARS-CoV-2 is African green monkey kidney Vero E6 cells, which express ACE2, but lack TMPRSS2 [6]. SARS-CoV-2 infection has been shown to be inefficient in respiratory-specific cell types [6,9,10], with suboptimal infection compared to Vero E6 cells. For most respiratory-specific cell types to be permissive to SARS-CoV-2 infection, artificial overexpression of ACE2 is required [10,11].

Interestingly, a recent study has shown that SARS-CoV-2 could infect the lung epithelial cell line H522 [9], which lacks both ACE2 and TMPRSS2, suggesting that entry into these cells was ACE2- and TMPRSS2-independent. One specific spike substitution that permitted infection into these cells was identified as substitution E484D, which is found in the RBD of the spike protein. Another study that examined cell-culture adaptation of a SARS-CoV-2 isolate in a different cell line also noted that the E484D substitution was important [12]. Furthermore, a previous study conducted by our lab described a SARS-CoV-2 variant adapted in human liver hepatoma (Huh7.5) cells with several spike protein changes (including E484D) that exhibited enhanced infectivity and replication in various human cell lines, and reduced dependency on entry via ACE2 [10]. However, given that these studies analyzed polyclonal viruses, which also had non-synonymous mutations in other viral proteins, a conclusive correlation between the changes in the spike protein and the forementioned phenotype is lacking. Pseudo-typing of SARS-CoV-2 is a useful tool to specifically investigate viral infectivity (entry) across different cell types, as investigation of mutations in the spike protein can be employed in a high throughput manner using a single infection event, reducing safety concerns and the need for high-level biosafety facilities. Furthermore, this tool provides data exclusively on the spike protein and not the other virus components, thereby focusing on viral entry [13].

In this study, a panel of SARS-CoV-2 pseudoparticles exhibiting various changes in the spike protein observed during cell culture adaptation of SARS-CoV-2 was investigated [10]. Infectivity assays were performed in different cell lines to deconvolute the specific spike protein changes affecting cell entry across cell lines. Furthermore, ACE2 entry dependence was characterized by using siRNA, a monoclonal anti-ACE2 antibody and drug blocking assays. Lastly, neutralization of the different SARS-CoV-2 pseudoparticles was performed using plasma from unvaccinated, convalescent SARS-CoV-2 infected individuals to understand if the cell culture adaptive spike protein changes affected neutralizing epitopes.

## Materials and methods

### Cell lines

Human embryonic kidney HEK293T cells, African Green Monkey kidney epithelial Vero E6 cells (kind gift from Prof. Jean Dubuisson), human hepatoma Huh7.5 cells (kind gift from Prof. Charles Rice) and human alveolar basal epithelial A549 cells with (Invivogen) and without (Sigma-Aldrich) overexpressed ACE2 were used for experiments. A549-ACE2 cells are transfected to express human ACE2 and are resistant to puromycin. All cell lines were grown in either 1x Dulbecco’s modified eagle medium (DMEM; Gibco) supplemented with 10% fetal bovine serum (FBS; HEK293T, Vero E6 and Huh7.5 cells), 1x DMEM/F-12 nutrient mixture (Gibco) supplemented with GlutaMAX (Gibco) and 10% FBS (A549 cells) or 1x F-12 Kaighn’s modified nutrient mixture (Gibco) supplemented with 10% FBS and 500 ug/mL puromycin (A549 + ACE2 cells). Cells were split a minimum of twice per week when they were observed to be between 70–90% confluent.

### Plasmids

A phCMV-5349 was used, encoding the murine leukemia virus (MLV) gag/pol, and pTG126, encoding the luciferase reporter (kind gift from Prof. Francois-Loic Cosset) [14]. Further, a pCG1 plasmid encoding the codon-optimized sequences for the spike glycoproteins of Wuhan-Hu-1 SARS-CoV-2 was used (kind gift from Markus Hoffmann) [6]. Finally, a phCMV plasmid encoding the hepatitis C virus (HCV) envelope (E1/E2) for Con1 was used (kind gift from Prof. Charles Rice) [15].

### Mutagenesis

To introduce culture-adapted mutations into the SARS-CoV-2 pCG1 plasmid, which carries the Wuhan-Hu-1 spike protein sequence, site-directed mutagenesis was performed. Initially, single amino acid substitutions at positions E309K and D614G were introduced to reproduce the spike glycoprotein of the DK-AHH1 isolate (GenBank accession number MZ049597). Following this, further mutagenesis was conducted to introduce the additional substitutions E484D, P812R, and Q954H using a two-step polymerase chain reaction (PCR) approach.

In the first step, a ‘megaprimer’ was generated via PCR. Reactions were set up in a 25 µl volume containing 12.5 µl of Q5 Hot Start High-Fidelity 2X Master Mix (New England Biolabs), 1.25 µl of 10 µM forward and reverse primers, 20–50 ng of template plasmid (DK-AHH1 pCG1), and nuclease-free water. PCR cycling involved an initial denaturation at 98°C for 30 seconds, followed by 30 cycles of 98°C for 10 seconds, 60°C for 10 seconds, and 72°C for 2 minutes, with a final extension at 72°C for 5 minutes. The resulting PCR products were treated with DPN1 enzyme (Thermo Fisher Scientific) for 2 hours at 37°C to digest the template plasmid, followed by gel electrophoresis for band recovery using a Zymoclean Gel DNA Recovery Kit (Zymo Research).

In the second step, the megaprimer was used in a 25 µl reaction containing 12.5 µl of Q5 Hot Start High-Fidelity 2X Master Mix, 300 ng of megaprimer, 100 ng of template plasmid, and nuclease-free water. PCR cycling conditions were as follows: 98°C for 30 seconds, followed by 20 cycles of 98°C for 10 seconds, 48°C for 1 minute, and 72°C for 35 minutes, with a final extension at 72°C for 35 minutes. The PCR products were treated with DPN1, analyzed by gel electrophoresis, and cleaned using a DNA Clean and Concentrator Kit (Zymo Research).

For the ∆68–76 spike variant, forward and reverse primers were used to create a linear PCR product omitting this region. The product was cleaned and re-circularized using a KLD enzyme mix (New England Biolabs) following the manufacturer’s instructions.

To generate the adapted spike protein containing all the mutations, a megaprimer was first generated to contain E484D, P812R and Q954H mutations. This megaprimer was then used on the ∆68–76 pCG1 plasmid to produce the plasmid containing all the culture-adapted mutations. A list of the spike protein mutants is provided in Table 1.

**Table 1:** Overview of the SARS-CoV-2 pseudoparticle panel.

Pseudoparticle name	Difference compared to Wuhan-Hu-1 spike
DK-AHH1	E309K & D614G
Δ68–76	Δ68–76, E309K & D614G
E484D	E309K, E484D & D614G
P812R	E309K, D614G & P812R
Q954H	E309K, D614G & Q954H
E484D+P812R	E309K, E484D, D614G & P812R
E484D+Q954H	E309K, E484D, D614G & Q954H
P812R+Q954H	E309K, D614G, P812R & Q954H
Δ68–76 + P812R+Q954H	Δ68–76, E309K, D614G, P812R & Q954H
E484D+P812R + Q954H	E309K E484D, D614G, P812R & Q954H
Adapted	Δ68–76, E309K, E484D, D614G, P812R & Q954H
VSV control	NA
HCV control (Con1, genotype 1b)	NA

All plasmids were sequence confirmed using Sanger sequencing (Macrogen Europe) and were transformed and grown in Top10 competent *E. coli* cells (Thermo Fisher Scientific) according to the manufacturer’s instructions. Large quantities of the plasmids were then obtained using a High-Speed Plasmid Maxi kit (Qiagen) according to the manufacturer’s instructions.

### Generation of pseudoparticles

Pseudoparticles were made by co-transfecting 5.5 µg phCMV-5349, 5.5 µg pTG126 and 2.5 µg pCG1 plasmid (Table 1) into 4x10^6^ HEK293T cells seeded in a 10 cm dish the day before using a mammalian Calphos transfection kit (Takara Bio) according to the manufacturer’s instructions. Successful incorporation of mutant spike proteins reflecting SARS-CoV-2 variants into the murine leukemia virus packaging vector (phCMV-5349) resulting in fully functional pseudo-typed viral particles has been previously validated using flow cytometry [16] and cryogenic electron microscopy [17]. Positive control pseudoparticles included a vesicular stomatitis virus (VSV) pseudoparticle and an HCV pseudoparticle. A negative control mock pseudoparticle was also generated by transfecting only phCMV-5349 and pTG126. Following transfection, the cells were incubated for 16−18 h at 37°C or 32°C and 5% CO_2_. The following day, media was replaced with fresh DMEM supplemented with 10% FBS without disrupting the cell layer and the cells were incubated at 37°C (HCV and VSV pseudoparticles) or 32°C (SARS-CoV-2 pseudoparticles) and 5% CO_2_ for 48 h. Following this, the supernatant was collected and purified of cellular debris by centrifugation at 500 x *g* for 10 min and the resulting supernatant poured into a fresh falcon tube. The purified supernatant was then mixed with polybrene (final concentration 4 µg/ml; Sigma-Aldrich) and added in quadruplicate to either 1x10^4^ Vero E6, Huh7.5, A549 or A549 + ACE2 cells seeded the day before in a 96-well white plate (Corning). The plates were then spinoculated for 2 h at 830 x *g* at 32°C. Following this, the plates were incubated for a further 2 h at 37°C (VSV and HCV pseudoparticles) or 32°C (SARS-CoV-2 pseudoparticles) and the pseudoparticle/polybrene mixture was aspirated from each well and replaced by prewarmed fresh media and incubated at 37°C (VSV and HCV pseudoparticles) or 32°C (SARS-CoV-2 pseudoparticles) and 5% CO_2_ for 72 h.

To measure the infectivity of the pseudoparticles, the media was aspirated, and the cells were lysed using a Glo Lysis Buffer (Promega) for 15 min on a plate rocker. Following this, an equal volume of Bright Glo reagent (Promega) was added and luminescence was detected using a FLUOstar Omega microplate reader (BMG Labtech). An infectious luminescence signal was determined as 10-fold higher than the mock pseudoparticle, lacking any spike or envelope glycoproteins. The signal/noise ratio was calculated as:


$$Signal/noise=\frac{Luminescence\;(pseudoparticle)}{Average\;luminescence\;(mock\;pseudoparticle)}$$

### Anti-ACE2, anti-TMPRSS2 and anti-TMEM106B antibody blocking assay

Vero E6, Huh7.5 and A549 cells were seeded on 96-well white plates (10^4^ cells/well) the day before. For blocking of ACE2, an anti-ACE2 antibody (R&D Systems, Cat#AF933) was added to the cells in quadruplicate at 20 µg/ml, 10 µg/ml, 1 µg/ml, 0.1 µg/ml, and 0.01 µg/ml for 2 h. For blocking of TMPRSS2, an anti-TMPRSS2 antibody (Thermo Fisher Scientific, Cat#PA5−14264) was added to the cells in quadruplicate at 50 µg/ml, 25 µg/ml, 12.5 µg/ml, 6.25 µg/ml and 3.125 µg/ml. Similarly, for blocking of TMEM106B, an anti-TMEM106B antibody (Thermo Fisher Scientific, Cat#60333–1-IG) was added to the cells in quadruplicate at 50 µg/ml, 25 µg/ml, 12.5 µg/ml, 6.25 µg/ml, and 3.125 µg/ml. Each pseudoparticle had 8 wells where phosphate buffered saline (PBS, Gibco) was added as non-treated controls. Following 2 h of incubation with the antibody, the media was aspirated from the wells and infection with the pseudoparticles was done as described above. The percentage infection was calculated as:


$$Infection\;(\;\%)=\frac{Luminescence\;(treated)}{Average\;luminescence\;(non-treated\;controls)}$$

If the average percentage infection of the treated quadruplicates was greater than 100% or less than 0%, the percentage infection was normalized to 100% and 0%, respectively.

### ACE2 siRNA assay

Vero E6, Huh7.5 and A549 cells were seeded on 96-well white plates (4 x 10^3^ cells/well) the day before. Cells were transfected with either 20 nM of scrambled siRNA (ON-TARGETplus non-targeting control, Horizon Discovery) or 20 nM ACE2 siRNA (ON-TARGETplus Human ACE2 siRNA, Horizon Discovery) using lipofectamine RNAiMAX (Thermo Fisher). At 48 h post-transfection, the cells were infected with pseudoparticles as described above. The percentage infection was calculated as described for the antibody blocking assays above.

### Drug treatment assays

Vero E6, Huh7.5 and A549 cells were seeded on 96-well white plates (10^4^ cells/well) the day before. The cells were either treated individually with aloxistatin (Sigma Aldich, Cat#E8640-250UG) at 100 µM, 10 µM, 1 µM, 0.1 µM and 0.01 µM or camostat mesylate (Sigma Aldich, Cat#SML0057−50MG) at 1000 µM, 100 µM, 10 µM, 1 µM and 0.1 µM, or treated in combination with aloxistatin (25 µM) and camostat mesylate (500 µM) together for 2 h. Each pseudoparticle had 8 wells where PBS (Gibco) was added as non-treated controls. Following the 2 h incubation, the media was aspirated, and the cells were infected with the pseudoparticles as described above. The percentage infection was calculated as described for the antibody blocking assays above.

### Neutralization assay

The neutralization assay was performed by adding 2-fold serially diluted convalescent plasma (starting at a 1:20 dilution and ranging up to a 1:640), collected from 6 individuals with confirmed SARS-CoV-2 infection that had non-hospitalized COVID-19 [10,18], to SARS-CoV-2 pseudoparticles in a 96 deep well plate and incubated for 1 h at room temperature. Pooled plasma from 5 healthy individuals with no previous exposure to SARS-CoV-2 was used as a comparative control. All participants were asked verbally about inclusion and signed an informed consent (Capital Region’s Committee on Health Research Ethics, project identifier H-20025872; Data Protection Agency (P-2020–357); samples collected from April to September 2020). Following this, the pseudoparticle/plasma mixture was then mixed with polybrene (final concentration 4 µg/ml; Sigma-Aldrich) and added in quadruplicate to either 1x10^4^ Vero E6, Huh7.5, A549, or A549 + ACE2 cells seeded the day before in a 96 well white plate. The plates were then spinoculated for 2 h at 830 x g at 32^o^C. Following this, the plates were incubated for a further 2 h at 32°C and the pseudoparticle/plasma/polybrene mixture was aspirated from each well and replaced by prewarmed fresh complete media and incubated at 32°C and 5% CO_2_ for 72 h. The percentage neutralization of the pseudoparticles was calculated as:


$$Neutralization\;(\;\%)=1-\left(\frac{Luminescence\;of\;pseudoparticle\;with\;convalescent\;plasma}{Average\;luminescence\;of\;pseudoparticle\;with\;healthy\;plasma}\right)x\;100$$

### Ethics and study approval

Plasma samples were obtained from the Clinical Virological and Immunological COVID-19 (CVIC) study [18–20]. This study has been approved by the Regional Ethical Committee (H-20025872) and the Data Protection Agency (P-2020–357) and was conducted in compliance with the Declaration of Helsinki guidelines. All individuals were enrolled on a volunteer basis and required to be at least 18 years of age and provide written informed consent. Collected study data was stored and managed using research electronic data capture (REDcap) tools hosted at Copenhagen University Hospital, Hvidovre [21].

### Statistics

All statistical tests were performed in GraphPad Prism (version 10.4.1). Comparisons of treated vs untreated experiments were done using multiple ratio paired t tests corrected for multiple comparisons using the Holm-Šídák method. 50% inhibitory dilution titers were calculated using non-linear regression (log[inhibitor] vs normalized response – variable slope). Comparisons of ID_50_ values was done using either a Mann-Whitney U test or a Kruskal-Wallis test corrected for multiple comparisons using Dunn’s test. All statistical tests used have been indicated in the text and figure legends. All statistical tests were two tailed. Statistical significance was defined by *p* < 0.0332 for the ratio paired t test and *p* < 0.05 for all other tests.

## Results

### Infectivity in different cell lines of SARS-CoV-2 pseudoparticles with cell-culture adaptive spike protein changes

In a prior study, serial passages of an ancestral-like SARS-CoV-2 isolate (DK-AHH1) were conducted in Huh7.5 cells [10]. Compared to the ancestral Wuhan-Hu-1 spike protein, DK-AHH1 harbored the E309K and D614G substitutions. Following multiple passages, the virus developed a cell-culture adapted phenotype with increased infectivity in various human cell lines, including those with low endogenous ACE2 expression levels. This cell culture adapted variant exhibited several spike protein changes, including E484D, P812R, Q954H, and a 9-amino acid deletion at positions 68–76 (Δ68–76). To investigate the impact of these spike alterations on viral entry, a panel of pseudoparticles containing individual or combined spike changes was engineered (Table 1). Entry was tested in multiple cell lines, including Vero E6 (green monkey kidney cells), Huh7.5 (human liver hepatoma cells), and A549 (a human lung carcinoma cell line with limited ACE2 expression). Additionally, A549 cells overexpressing ACE2 (A549-ACE2) were included. For controls, pseudoparticles based on VSV and HCV (genotype 1b, isolate Con1) were used across all cell lines [6,22].

In the pseudoparticle panel, the spike protein of the adapted isolate, containing the combination of Δ68–76, E484D, P812R, and Q954H, was referred to as “adapted.” Positive infection was defined as luminescence exceeding 10-fold relative to mock pseudoparticles lacking spike. Pseudoparticles with VSV, or HCV envelope proteins were included as controls.

**Fig 1:**
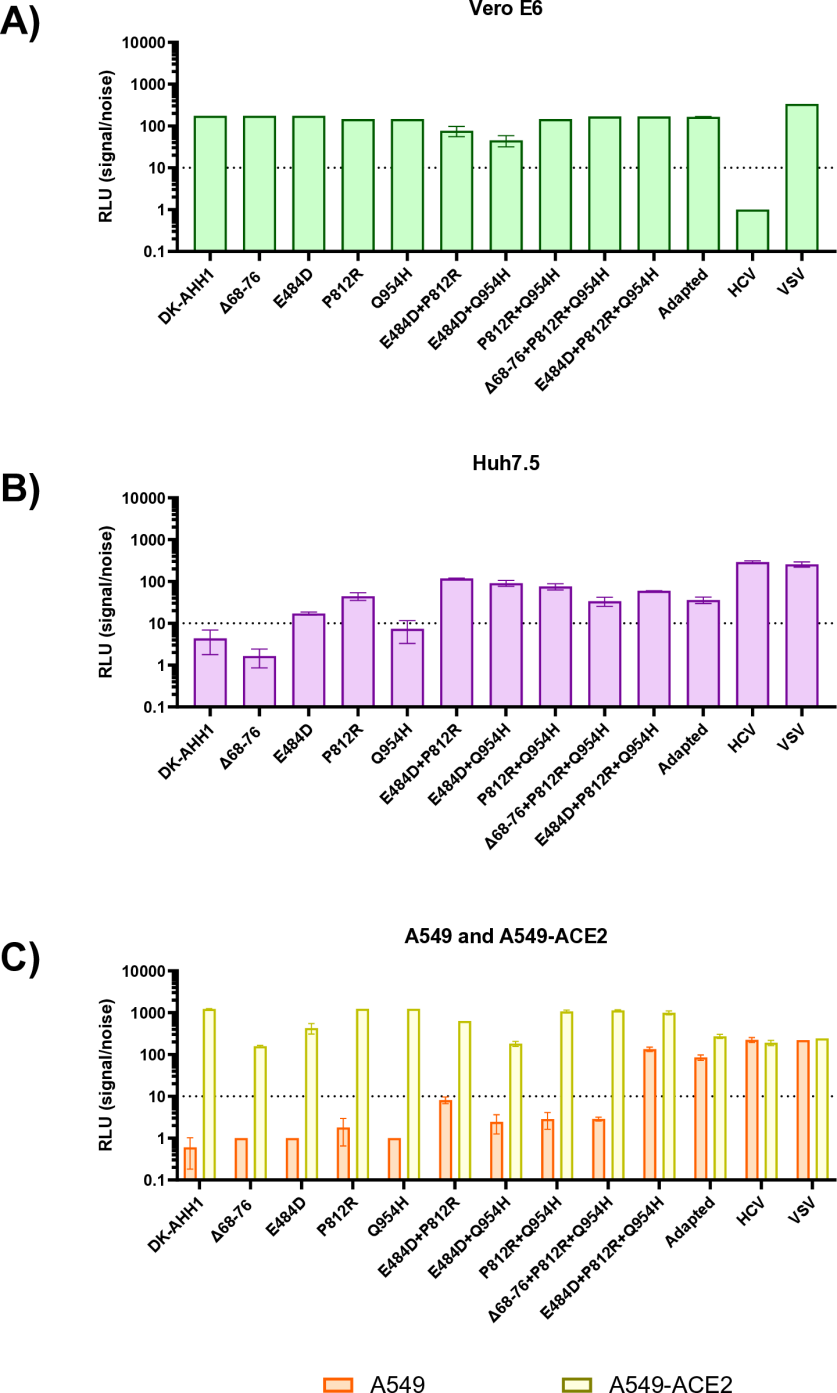
Infectivity of SARS-CoV-2 pseudoparticle panel across multiple cell lines. A panel of pseudoparticles carrying different spike protein changes was measured for infectivity in Vero E6 cells (**A**, green), Huh7.5 cells (**B**, purple), A549 cells (**C**, orange) and A549 cell overexpressing ACE2 (A549-ACE2, **C**, yellow). Infectivity is displayed as signal/noise (S/N) as luminescence of the indicated pseudoparticle compared to luminescence of the mock pseudoparticle (S1 Fig). A positive signal was determined as 10-fold higher luminescence than the mock pseudoparticle (dotted line). Pseudoparticles under the dotted line were deemed non-infectious and were not continued for further experiments. The error bars represent the standard deviation from the recorded luminescence of the four pseudoparticle replicates. The reported mean and standard deviations for Fig 1A–1C can be found in S1 Table.

In Vero E6 cells, all pseudoparticles, except the HCV pseudoparticle, were found to be highly infectious (Fig 1A). In Huh7.5 cells, all pseudoparticles were infectious, except for WT, Δ68–76, and Q954H pseudoparticles (Fig 1B). Notably, pseudoparticles carrying either E484D or P812R alone conferred permissive infection, and their combination further increased infectivity. However, the addition of Δ68–76 or Q954H to the pseudoparticle carrying both E484D and P812R substitutions (E484D+P812R) did not increase infectivity further. Interestingly, the Q954H substitution combined with either E484D (E484D+Q954H) or P812R (P812R+Q954H) showed higher infectivity than pseudoparticles carrying either E484D or P812R alone. In A549 cells, only the pseudoparticle carrying the combined E484D, P812R and Q954H substitutions (E484D+P812R + Q954H) and the adapted pseudoparticle, along with the HCV and VSV pseudoparticles, were infectious (Fig 1C), suggesting that all three substitutions, but not Δ68–76, are necessary for permissive entry into A549 cells. However, when ACE2 was overexpressed in A549 cells, all pseudoparticles became infectious, indicating that ACE2 overexpression can rescue infectivity in these cells.

### Exploring the dependency of ACE2 for SARS-CoV-2 cell entry

Multiple studies have demonstrated that specific spike protein changes enable SARS-CoV-2 to enter cells independently of ACE2 [9,23,24]. To investigate the effect of the spike protein changes on the pseudoparticle’s dependency of ACE2 for entry, ACE2 blocking was performed using three independent approaches: blocking with an anti-ACE2 antibody, silencing ACE2 expression using siRNA, and inhibiting cathepsin-mediated entry with aloxistatin. These methods were applied to all pseudoparticles found to be infectious in each cell line. Blocking via anti-ACE2 antibody or aloxistatin was done in a dose-dependent manner (S2 Fig).

**Fig 2:**
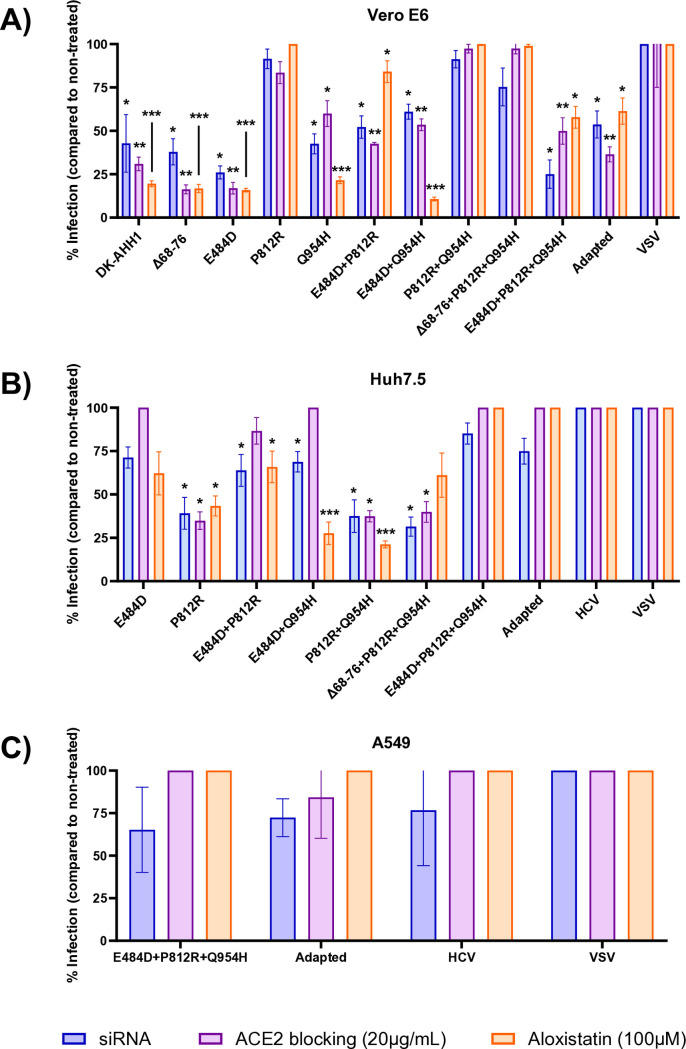
Infectivity of the pseudoparticles in different cell lines following blocking of ACE2. Blocking of ACE2 was done using siRNA (blue), anti-ACE2 antibody (purple) or aloxistatin (orange) in Vero E6 (**A**), Huh7.5 (**B**) or A549 (**C**) cells. Blocking of ACE2 via anti-ACE2 antibody and aloxistatin was done in a dose-dependent manner (S2 Fig) but the highest concentration used is shown here. Blocking is shown as the percentage infection compared to the non-treated control. If the average of the quadruplicates was over 100% infectivity compared to the non-treated control, the data was normalized to 100%. The error bars represent the standard deviation from the calculated percentage infection of the four pseudoparticle replicates. The reported means and standard deviations for Fig 2A–2C can be found in S2–S4 Tables, respectively. Comparisons between untreated and treated experiments were done using multiple ratio paired t tests corrected for multiple comparisons using the Holm-Šídák method. *p < 0.0332, **p < 0.0021, ***p < 0002.

In Vero E6 cells, pseudoparticles containing the P812R substitution were found to exhibit high levels of infectivity across all ACE2-blocking assays (Fig 2A). However, pseudoparticles carrying both E484D and P812R showed increased entry dependency on ACE2 compared to pseudoparticles lacking the E484D substitution, suggesting the E484D substitution may increase entry dependency on ACE2 in Vero E6 cells. In Huh7.5 cells, E484D was identified as the key driver of ACE2-independent entry, as pseudoparticles containing this substitution remained infectious under all ACE2-blocking conditions and pseudoparticles without E484D were significantly affected by ACE2 blocking in all conditions (Fig 2B). In A549 cells, both the E484D+P812R + Q954H pseudoparticle and the adapted pseudoparticle exhibited high infectivity despite ACE2 blocking (Fig 2C), indicating that these pseudoparticles can enter A549 cells through an ACE2-independent mechanism.

### Exploring SARS-CoV-2 pseudoparticle entry dependency of TMPRSS2 and TMEM106B

Since TMPRSS2 has been identified as an important entry receptor for SARS-CoV-2, blocking of this receptor was performed in a dose-dependent manner using both an anti-TMPRSS2 antibody and the serine protease inhibitor camostat. Although Vero E6 cells do not express TMPRSS2, this cell line was included as a control in the blocking assays. No inhibition of infection was observed in any cell line at the highest concentrations of the anti-TMPRSS2 antibody or camostat (S3 Fig).

**Fig 3:**
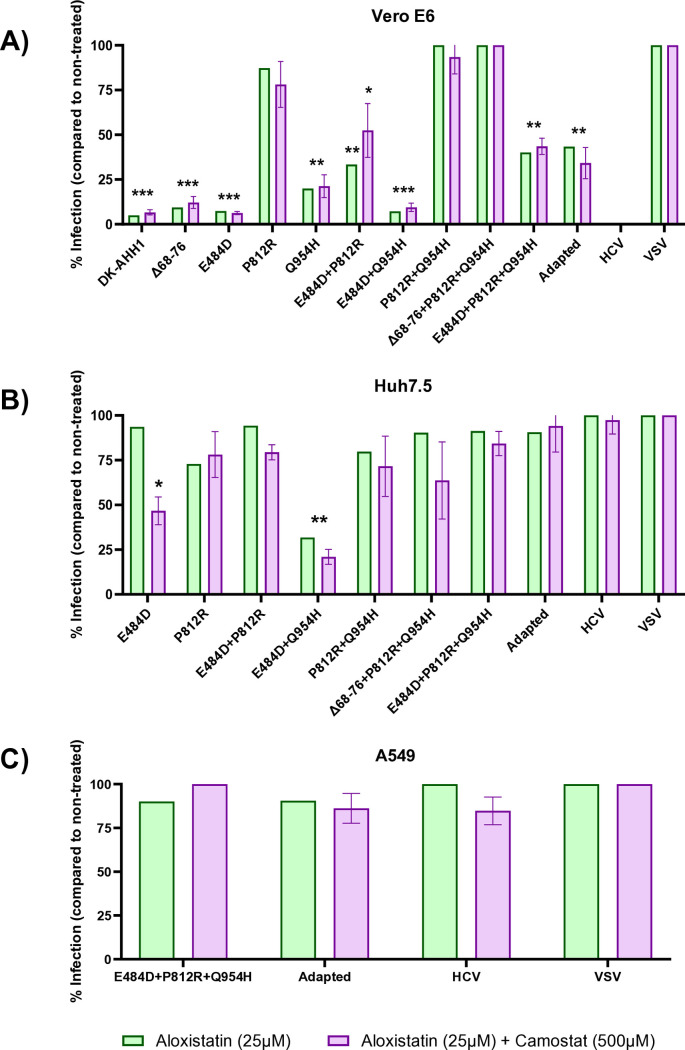
Infectivity of the pseudoparticles following treatment of aloxistatin and camostat mesylate in different cell lines. Vero E6 (**A**), Huh7.5 (**B**) or A549 (**C**) cells were treated with aloxistatin (25µM) and camostat mesylate (500µM, purple) and compared to aloxistatin treatment alone (25µM, green). The singular treatment of aloxistatin at 25µM was interpolated from the dose-dependent treatment done (S2 Fig). Infection of the pseudoparticles was compared to the non-treated control. As treatment with camostat mesylate alone did not show any inhibition of infection for any pseudoparticle (S3 Fig), this data was not presented here. If the average of the quadruplicates was over 100% infectivity compared to the non-treated control, the data was normalized to 100%. The error bars represent the standard deviation from the calculated percentage infection of the four pseudoparticle replicates. The reported means and standard deviations for Fig 3A–3C can be found in S5–S7 Tables, respectively. Comparisons between untreated and treated experiments were done using multiple ratio paired t tests corrected for multiple comparisons using the Holm-Šídák method. *p < 0.0332, **p < 0.0021, ***p < 0002.

To assess whether blocking both ACE2 and TMPRSS2 would enhance inhibition compared to ACE2 blockade alone, based on a prior study [6], a single concentration of aloxistatin (25 µM) and camostat (500 µM) was used to block each pseudoparticle in all cell lines. To enable comparison with aloxistatin alone, inhibition levels at 25 µM were interpolated from the previously performed dose-dependent blocking of aloxistatin (S2 Fig). Except for the E484D pseudoparticle in Huh7.5 cells, the addition of camostat did not further inhibit pseudoparticle entry for any tested pseudoparticle (Fig 3), suggesting that entry independent of ACE2 is also independent of TMPRSS2 when E484D and P812R are combined.

Lastly, as previous reports indicated that TMEM106B could act as an alternative receptor for SARS-CoV-2 pseudoparticles in a cell line lacking ACE2 [23], blocking of TMEM106B was performed using an anti-TMEM106B antibody for the E484D+P812R + Q954H and adapted pseudoparticles across all cell lines. However, no inhibition of infection was observed for any pseudoparticle (S3 Fig).

### Comparison of neutralization of the WT and adapted SARS-CoV-2 pseudoparticles using COVID-19 convalescent plasma

**Fig 4:**
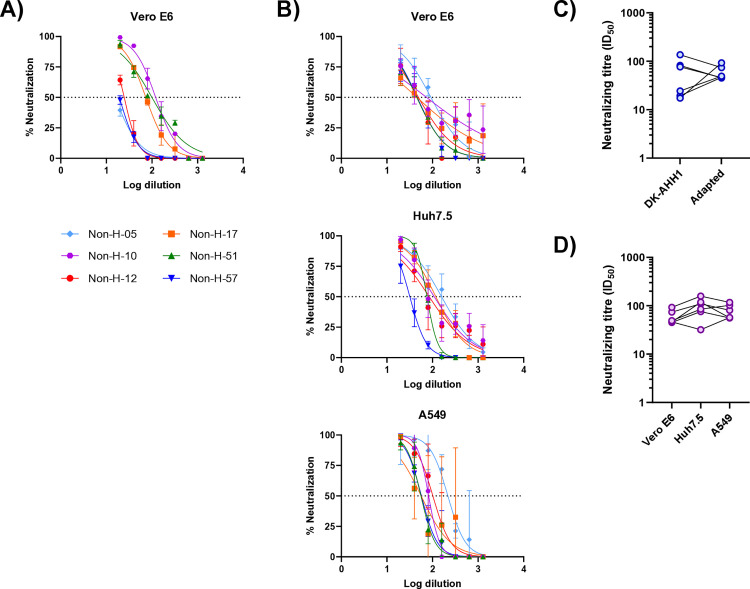
Neutralization of the DK-AHH1 and adapted pseudoparticles. Neutralization curves of 6 convalescent plasma samples against the DK-AHH1 (**A**) and adapted (**B**) pseudoparticles in the indicated cell lines. Error bars represent the standard deviation. The dotted line represents the 50% neutralization level. The neutralizing antibody titers (ID_50_s) of the DK-AHH1 and adapted pseudoparticles were compared in Vero E6 cells with no significant differences detected (**C**, Mann-Whitney U test). Similarly, the neutralizing titers (ID_50_s) of the adapted pseudoparticle were compared across cell lines with no significant differences detected (**D**, Kruskal-Wallis test). The error bars represent the standard deviation from the calculated percentage neutralization of the four pseudoparticle replicates. The reported means and standard deviations for Fig 4A can be found in S8 Table. The reported means and standard deviations for Fig 4B can be found in S9–S11 Tables. The calculated ID_50_ values for Fig 4C and 4D can be found in S12 Table.

To assess whether the spike protein changes that enhanced infectivity in different cell lines also affected neutralization sensitivity, neutralization experiments were conducted using convalescent plasma from six unvaccinated individuals who had recovered from SARS-CoV-2 infection. This plasma panel had previously been used to evaluate neutralization of the cell-culture adapted SARS-CoV-2 virus variant in Vero E6 cells [10,18]. Due to the low infectivity of the WT pseudoparticle in other cell lines (Fig 1A), neutralization of the WT pseudoparticle was only performed in Vero E6 cells (Fig 4A). For the adapted pseudoparticle, neutralization was assessed in all cell lines (Fig 4B).

First, 50% inhibitory dilutions (ID_50_s) were compared between the WT and adapted pseudoparticles in Vero E6 cells to determine whether the spike protein changes in the adapted pseudoparticle led to a different neutralization profile (Fig 4C). However, no significant difference was observed (p > 0.05, Mann-Whitney U test). Next, ID_50_s for the adapted SARS-CoV-2 pseudoparticle were compared across the different cell lines to evaluate whether neutralization was consistent across cell types (Fig 4D), and no significant differences in ID_50_s were found (p > 0.05, Kruskal-Wallis test).

## Discussion

Despite ongoing vaccinations, preventative measures and therapies, SARS-CoV-2 remains a global health threat. Throughout the pandemic, SARS-CoV-2 gradually accumulated spike protein changes that have been linked to changes in infectivity, transmissibility, and immune evasion. Several studies have shown that cell culture-adapted SARS-CoV-2 isolates harbor spike protein changes that improve infectivity in different cell lines, often with reduced dependency on ACE2 for entry [9,10,24]. The present study evaluated the infectivity, ACE2 entry dependency and neutralization phenotype of pseudoparticles with spike protein changes identified previously that were involved in enhancing entry of SARS-CoV-2 in human cell lines [10], either individually or in different combinations. The benefit of this approach is that it allows specific assessment of the spike protein without the other SARS-CoV-2 constituents. The results in this study revealed how key spike protein alterations, such as E484D, P812R and Q954H can modulate SARS-CoV-2 entry mechanisms and alter infectivity profiles across different cell lines. Importantly, the observations in our previous study, whereby the cell culture-adapted SARS-CoV-2 isolate led to enhanced viability across human cell lines, including Huh7.5 and A549 cells, were recapitulated here in the pseudovirus model. In addition, this study also validated the reduced dependency of ACE2 for entry in these cell lines, with no changes in neutralization by convalescent plasma from individuals with COVID-19.

By isolating individual spike protein changes, it was revealed here that the E484D and P812R spike substitutions play critical, yet distinct, roles in mediating infectivity and ACE2-independent entry in different cell lines. In Vero E6 cells, while all the pseudoparticles were found to be infectious, the P812R emerged as the primary driver for ACE2-independent entry as any SARS-CoV-2 pseudoparticles lacking this substitution were susceptible to inhibition by ACE2-blocking reagents. Interestingly, when E484D was included with P812R, a higher dependency on ACE2 for entry was observed as infectivity was more inhibited by ACE2-blocking reagents compared to pseudoparticles carrying P812R alone.

In Huh7.5 cells, which have minimal endogenous ACE2 expression [25], DK-AHH1, Δ68−76 and Q954H pseudoparticles were not found to be infectious. Pseudoparticles carrying E484D or P812R, either individually or in combination with other spike protein changes, permitted infection. Interestingly, E484D was the primary driver for ACE2-independent entry in Huh7.5 cells as any SARS-CoV-2 pseudoparticles lacking this substitution were affected by ACE2-blocking reagents. Interestingly, in another study exploring SARS-CoV-2 entry, E484D was found to drive entry efficiency in Huh7 cells but not in other cell types, such as A549 cells [26]. In the present study, the combination of P812R or Q954H with E484D (E484D+P812R or E484D+Q954H) did not alter this phenotype greatly, however, the triple combination of E484D, P812R and Q954H further enhanced ACE2-independent entry. These results suggest that E484D’s effects on the entry dependency for ACE2 are context-dependent, varying with the specific cell type and its receptor expression profile. The fact that P812R mediated ACE2-independent entry in Vero E6 cells but not Huh7.5 cells underscores the versatility of SARS-CoV-2 in adapting its entry strategy.

A549 cells, which also express ACE2 in low amounts [27], have been reported to be non-permissive to SARS-CoV-2 infection [28], but permissive for HCV infection [29,30]. However, it has been demonstrated that the E484D substitution can enable infectivity in these cells [9,24]. In the present study, as well as another study [26], E484D alone was not enough to permit detectable entry in A549 cells. Instead, all three substitutions, but not the Δ68–76 deletion, were required for permissive pseudoparticle infection. Interestingly, serial passage of SARS-CoV-2 in A549 cells has been shown to give rise to, among others, E484D, as well as a P812L substitution [31], suggesting that these two residues are important for cell culture adaptation of SARS-CoV-2 in A549 cells. In the present study, blocking of ACE2 for both the E484D+P812R + Q954H and adapted pseudoparticles did not show any major changes in infectivity, suggesting that entry was done in an ACE2-independent fashion. Interestingly, the Δ68–76 deletion was not found to make any significant differences to infectivity or ACE2-independent entry for any cell line as the adapted pseudoparticle was highly comparable to the E484D+P812R + Q954H pseudoparticle, which lacked the Δ68–76 deletion. These findings suggest that E484D, P812R and Q954H in combination are critical for permitting entry into multiple cell lines in an ACE2-independent fashion. However, the presence of the Δ68–76 deletion may still contribute to viral fitness in specific contexts or cell types that were not explored in this study.

In other studies assessing ACE2-independent entry for SARS-CoV-2, the E484D substitution has been shown to significantly enhance the entry of SARS-CoV-2 in cells lacking expression of both ACE2 and TMPRSS2 [9,23,24]. Furthermore, E484D has been linked to adaptation to cells expressing murine ACE2 [32], permitting infection into cells lacking ACE2 expression including murine, hamster and porcine cell lines [32], further highlighting its importance for ACE2-indepenent entry. Possible alternative entry receptors have been linked to TMEM106B and heparin sulfates [23,33,34]. However, in this study, blocking of TMEM106B using an anti-TMEM106B antibody was not found to inhibit infection, which may be due to the different cell lines used. While E484D wasn’t completely necessary for entry into Huh7.5 cells in this study, it has been found to be important for ACE2-independent entry in Huh7 cells by others [24]. Structurally, the E484D substitution has been proposed to induce a retraction of the carboxyl group positioned between Y489 and F490 [32], potentially altering spike-receptor interactions. In a recent study by our group [35], a spike RBD protein carrying the E484D substitution was found to dissociate from ACE2 faster than E484 or other E484 substitutions. For cells expressing low levels of ACE2, this faster dissociation from ACE2 may allow the spike protein to engage with alternative receptors, promoting ACE2-independent entry.

The P812R substitution, located downstream from the furin cleavage site (P681-R685), has been proposed to form a second cleavage site [10], which may provide an added benefit for fusion of the virus and host membranes. Interestingly, although P812R emerged during adaptation in Huh7.5 cells [10], it was found to be beneficial for ACE2-independent entry in Vero E6 cells. Given that Vero E6 cells lack TMPRSS2 and that aloxistatin, which blocks cathepsin-mediated entry, did not greatly affect the P812R pseudoparticle, it is possible that this substitution permits direct fusion of the viral and host membranes. However, this entry mechanism is likely to be specific to Vero E6 cells as this was not seen in Huh7.5 or A549 cells. Given that camostat, which blocks serine protease activity, did not affect infectivity of the P812R pseudoparticle in both Vero E6 and Huh7.5 cells, fusion of the viral and host membranes was likely done independently of serine protease activity. As Huh7 and A549 cells have low levels of both endogenous ACE2 and TMPRSS2 [24,26,36], this may explain why adaptation of SARS-CoV-2 isolates in these cells leads to a substitution at P812, whereas adaptation in cells overexpressing TMPRSS2, do not give rise to this mutation [37]. Interestingly, adaptation of SARS-CoV-2 in VeroE6 cells has been shown to increase entry efficiency by improving cleavage of the spike protein by cathepsins, possibly by altering the furin cleavage site, but decreased entry efficiency in TMPRSS2^+^ cell lines [37,38]. Together, this highlights that cell-type-specific protease expression strongly influences SARS-CoV-2 cell culture adaptation. Other studies exploring this area of the spike protein have shown that substituting P812S decreased the binding affinity of the spike protein to TMPRSS2 [39], and substituting T813S, which is located adjacent to position 812, led to decreased cleavage of the spike protein by TMPRSS2 [40]. Collectively, this would suggest that substitutions at or around the P812 site likely mediate cleavage of the spike protein independently of serine proteases, such as TMPRSS2. As blocking of serine protease activity via camostat did not show any differences in infectivity for all pseudoparticles in all cell lines, it is highly possible that entry is granted independently of any serine proteases. This was further confirmed when blocking was performed with the combination of aloxistatin and camostat, where only the E484D pseudoparticle, and possibly the E484D+Q954H pseudoparticle, in Huh7.5 cells were found to have reduced infectivity compared to blocking with aloxistatin alone. The fact that these two pseudoparticles lacked the P812R substitution further highlights the importance of this substitution for entry independent of serine proteases.

The Q954H substitution is located in the heptad repeat 1 (HR1) region, which is a region located in the S2 subunit important for stability and fusion of the spike protein to the target cell membrane [41]. This specific substitution is also found to be fixed within the omicron lineages of SARS-CoV-2, highlighting its importance [42]. In this study, no major changes in infectivity or ACE2 entry independency were observed for Q954H. However, when combined with either E484D or P812R, enhancement of infection was seen in Huh7.5 cells. Furthermore, without this substitution, entry was not possible in A549 cells, suggesting that this substitution in combination with E484D and P812R is important for infectivity in different cell lines and for entry in an ACE2-independent fashion. While the exact role of Q954H for entry is not clear, it is possible that this substitution may increase the fusogenicity of the viral membrane to the host membranes.

Although previous assessment of neutralization of the DK-AHH1 and adapted isolates was compared [10], it was also important to assess this in the pseudo-typed model as this model only contains the spike protein, meaning that antibodies directed to other proteins are not able to influence neutralization. Furthermore, previous assessment of neutralization was only done in Vero E6 cells and, given that the adapted spike protein can infect other cell lines that express different receptors, it was important to see if neutralization across different cell lines was comparable. When the DK-AHH1 and adapted pseudoparticles were compared, no significant differences were found in the neutralizing titers, further confirming that the spike protein changes found in the adapted isolate do not alter neutralizing epitopes. When comparing the neutralizing titers of the adapted isolate across different cell lines, they were also found to be comparable, suggesting that, despite there being different receptors expressed across the cell lines, neutralization of the virus is conserved.

## Conclusion

In summary, this study identifies key spike protein changes that enable infection into multiple cell lines in an ACE2- and serine protease-independent manner. While the exact mechanisms behind ACE2- and serine protease-independent entry were not elucidated, the distinct roles of E484D and P812R in mediating ACE2- and serine protease-independent entry underscores the virus’s ability to adapt to utilize alternative receptors or pathways, ensuring robust infectivity across various cell types. These insights deepen our understanding of how SARS-CoV-2 evolves to evade receptor-specific barriers and may inform on future therapeutic strategies aimed at targeting viral entry mechanisms. Furthermore, this study warrants the exploration of alternative entry mechanisms employed by SARS-CoV-2.

## Supporting information

S1 Fig Raw infectivity of each pseudoparticle (in relative luminescence units [RLU]) in VeroE6 cells showing the relative luminescence output compared to the mock pseudoparticle lacking spike or envelope proteins. (TIF)

S2 Fig Infectivity of the pseudoparticles following dose-dependent treatment of Vero E6 (Top), Huh7.5 (middle) and A549 (bottom) with anti-ACE2-antibody (left) and aloxistatin (right). The error bars represent the standard deviation from the calculated percentage infection of the four pseudoparticle replicates. All calculated 50% inhibitory values can be found in S13 Table.
(TIF)

S3 Fig Infectivity of the pseudoparticles following dose-dependent treatment of Vero E6 (left), Huh7.5 (middle) and A549 (right) with anti-TMRPSS2-antibody (top), camostat mesylate (middle) and anti-TMEM106B antibody (bottom). The error bars represent the standard deviation from the calculated percentage infection of the four pseudoparticle replicates.
(TIF)

S1 Table Infectivity of each pseudoparticle measured as signal/noise in relative luminescence units (RLUs) plotted in Fig 1. (DOCX)

S2 Table The percentage (%) infection values (compared to the non-treated control) plotted in Fig 2A (Vero E6 cells). (DOCX)

S3 Table The percentage (%) infection values (compared to the non-treated control) plotted in Fig 2B (Huh7.5 cells). (DOCX)

S4 Table The percentage (%) infection values (compared to the non-treated control) plotted in Fig 2C (A549 cells). (DOCX)

S5 Table The percentage (%) infection values (compared to the non-treated control) plotted in Fig 3A (Vero E6 cells). (DOCX)

S6 Table The percentage (%) infection values (compared to the non-treated control) plotted in Fig 3B (Huh7.5 cells). (DOCX)

S7 Table The percentage (%) infection values (compared to the non-treated control) plotted in Fig 3C (A549 cells). (DOCX)

S8 Table The percentage (%) neutralization values for neutralization of DK-AHH1 in Vero E6 cells (Fig 4A). (DOCX)

S9 Table The percentage (%) neutralization values for neutralization of the adapted variant in Vero E6 cells (Fig 4B). (DOCX)

S10 Table The percentage (%) neutralization values for neutralization of the adapted variant in Huh7.5 cells (Fig 4B). (DOCX)

S11 Table The percentage neutralization values for neutralization of the adapted variant in A549 cells (Fig 4B). (DOCX)

S12 Table ID_50_ values for the DK-AHH1 and adapted pseudoparticle across cell lines. (DOCX)

S13 Table IC_50_ (anti-ACE2 antibody) and EC_50_ (aloxistatin) values for the panel of pseudoparticle across cell lines. (DOCX)

## References

[1] World Health Organization COVID-19 overview. [cited 2023 Aug]. Available from: https://covid19.who.int.}

[2] Yang Z, Zhang S, Tang Y-P, Zhang S, Xu D-Q, Yue S-J, et al. Clinical characteristics, transmissibility, pathogenicity, susceptible populations, and re-infectivity of prominent COVID-19 variants. Aging Dis. 2022;13(2):402–22. doi: 10.14336/AD.2021.1210 35371608 PMC8947836

[3] Dickey TH, Tang WK, Butler B, Ouahes T, Orr-Gonzalez S, Salinas ND, et al. Design of the SARS-CoV-2 RBD vaccine antigen improves neutralizing antibody response. Sci Adv. 2022;8(37):eabq8276. doi: 10.1126/sciadv.abq8276 36103542 PMC9473567

[4] Huang Y, Yang C, Xu X-F, Xu W, Liu S-W. Structural and functional properties of SARS-CoV-2 spike protein: potential antivirus drug development for COVID-19. Acta Pharmacol Sin. 2020;41(9):1141–9. doi: 10.1038/s41401-020-0485-4 32747721 PMC7396720

[5] Li M-Y, Li L, Zhang Y, Wang X-S. Expression of the SARS-CoV-2 cell receptor gene ACE2 in a wide variety of human tissues. Infect Dis Poverty. 2020;9(1):45. doi: 10.1186/s40249-020-00662-x 32345362 PMC7186534

[6] Hoffmann M, Kleine-Weber H, Schroeder S, Krüger N, Herrler T, Erichsen S, et al. SARS-CoV-2 cell entry depends on ACE2 and TMPRSS2 and is blocked by a clinically proven protease inhibitor. Cell. 2020;181(2):271-280.e8. doi: 10.1016/j.cell.2020.02.052 32142651 PMC7102627

[7] Liu J, Li Y, Liu Q, Yao Q, Wang X, Zhang H, et al. SARS-CoV-2 cell tropism and multiorgan infection. Cell Discov. 2021;7(1):17. doi: 10.1038/s41421-021-00249-2 33758165 PMC7987126

[8] Puelles VG, Lütgehetmann M, Lindenmeyer MT, Sperhake JP, Wong MN, Allweiss L, et al. Multiorgan and renal tropism of SARS-CoV-2. N Engl J Med. 2020;383(6):590–2. doi: 10.1056/NEJMc2011400 32402155 PMC7240771

[9] Puray-Chavez M, LaPak KM, Schrank TP, Elliott JL, Bhatt DP, Agajanian MJ, et al. Systematic analysis of SARS-CoV-2 infection of an ACE2-negative human airway cell. Cell Rep. 2021;36(2):109364. doi: 10.1016/j.celrep.2021.109364 34214467 PMC8220945

[10] Ramirez S, Fernandez-Antunez C, Galli A, Underwood A, Pham LV, Ryberg LA, et al. Overcoming culture restriction for SARS-CoV-2 in human cells facilitates the screening of compounds inhibiting viral replication. Antimicrob Agents Chemother. 2021;65(7):e0009721. doi: 10.1128/AAC.00097-21 33903110 PMC8406809

[11] Xie X, Muruato AE, Zhang X, Lokugamage KG, Fontes-Garfias CR, Zou J, et al. A nanoluciferase SARS-CoV-2 for rapid neutralization testing and screening of anti-infective drugs for COVID-19. Nat Commun. 2020;11(1):5214. doi: 10.1038/s41467-020-19055-7 33060595 PMC7567097

[12] Szemiel AM, Merits A, Orton RJ, MacLean OA, Pinto RM, Wickenhagen A, et al. In vitro selection of Remdesivir resistance suggests evolutionary predictability of SARS-CoV-2. PLoS Pathog. 2021;17(9):e1009929. doi: 10.1371/journal.ppat.1009929 34534263 PMC8496873

[13] Havranek KE, Jimenez AR, Acciani MD, Lay Mendoza MF, Reyes Ballista JM, Diaz DA, et al. SARS-CoV-2 spike alterations enhance pseudoparticle titers and replication-competent VSV-SARS-CoV-2 virus. Viruses. 2020;12(12):1465. doi: 10.3390/v12121465 33353101 PMC7767099

[14] Bartosch B, Dubuisson J, Cosset F-L. Infectious hepatitis C virus pseudo-particles containing functional E1-E2 envelope protein complexes. J Exp Med. 2003;197(5):633–42. doi: 10.1084/jem.20021756 12615904 PMC2193821

[15] Prentoe J, Serre SBN, Ramirez S, Nicosia A, Gottwein JM, Bukh J. Hypervariable region 1 deletion and required adaptive envelope mutations confer decreased dependency on scavenger receptor class B type I and low-density lipoprotein receptor for hepatitis C virus. J Virol. 2014;88(3):1725–39. doi: 10.1128/JVI.02017-13 24257605 PMC3911595

[16] Roy S, Ghani K, de Campos-Lima PO, Caruso M. A stable platform for the production of virus-like particles pseudotyped with the severe acute respiratory syndrome coronavirus-2 (SARS-CoV-2) spike protein. Virus Res. 2021;295:198305. doi: 10.1016/j.virusres.2021.198305 33482242 PMC7817443

[17] Petersen JD, Lu J, Fitzgerald W, Zhou F, Blank PS, Matthies D, et al. Unique aggregation of retroviral particles pseudotyped with the delta variant SARS-CoV-2 spike protein. Viruses. 2022;14(5):1024. doi: 10.3390/v14051024 35632764 PMC9147488

[18] Underwood AP, Sølund C, Fernandez-Antunez C, Villadsen SL, Winckelmann AA, Bollerup S, et al. Neutralisation titres against SARS-CoV-2 are sustained 6 months after onset of symptoms in individuals with mild COVID-19. EBioMedicine. 2021;71:103519. doi: 10.1016/j.ebiom.2021.103519 34419923 PMC8375401

[19] Sølund C, Underwood AP, Fernandez-Antunez C, Bollerup S, Mikkelsen LS, Villadsen SL, et al. Analysis of neutralization titers against SARS-CoV-2 in health-care workers vaccinated with Prime-Boost mRNA-mRNA or Vector-mRNA COVID-19 vaccines. Vaccines (Basel). 2022;10(1):75. doi: 10.3390/vaccines10010075 35062736 PMC8780959

[20] Underwood AP, Sølund C, Fernandez-Antunez C, Villadsen SL, Mikkelsen LS, Fahnøe U, et al. Durability and breadth of neutralisation following multiple antigen exposures to SARS-CoV-2 infection and/or COVID-19 vaccination. EBioMedicine. 2023;89:104475. doi: 10.1016/j.ebiom.2023.104475 36870117 PMC9978324

[21] Harris PA, Taylor R, Thielke R, Payne J, Gonzalez N, Conde JG. Research electronic data capture (REDCap)--a metadata-driven methodology and workflow process for providing translational research informatics support. J Biomed Inform. 2009;42(2):377–81. doi: 10.1016/j.jbi.2008.08.010 18929686 PMC2700030

[22] Schaller T, Appel N, Koutsoudakis G, Kallis S, Lohmann V, Pietschmann T, et al. Analysis of hepatitis C virus superinfection exclusion by using novel fluorochrome gene-tagged viral genomes. J Virol. 2007;81(9):4591–603. doi: 10.1128/JVI.02144-06 17301154 PMC1900174

[23] Baggen J, Jacquemyn M, Persoons L, Vanstreels E, Pye VE, Wrobel AG, et al. TMEM106B is a receptor mediating ACE2-independent SARS-CoV-2 cell entry. Cell. 2023;186(16):3427-3442.e22. doi: 10.1016/j.cell.2023.06.005 37421949 PMC10409496

[24] Hoffmann M, Sidarovich A, Arora P, Krüger N, Nehlmeier I, Kempf A, et al. Evidence for an ACE2-independent entry pathway that can protect from neutralization by an antibody used for COVID-19 therapy. mBio. 2022;13(3):e0036422. doi: 10.1128/mbio.00364-22 35467423 PMC9239067

[25] Ren X, Glende J, Al-Falah M, de Vries V, Schwegmann-Wessels C, Qu X, et al. Analysis of ACE2 in polarized epithelial cells: surface expression and function as receptor for severe acute respiratory syndrome-associated coronavirus. J Gen Virol. 2006;87(Pt 6):1691–5. doi: 10.1099/vir.0.81749-0 16690935

[26] Carrascosa-Sàez M, Marqués M-C, Geller R, Elena SF, Rahmeh A, Dufloo J, et al. Cell type-specific adaptation of the SARS-CoV-2 spike. Virus Evol. 2024;10(1):veae032. doi: 10.1093/ve/veae032 38779130 PMC11110937

[27] Chang C-W, Parsi KM, Somasundaran M, Vanderleeden E, Liu P, Cruz J, et al. A newly engineered A549 cell line expressing ACE2 and TMPRSS2 is highly permissive to SARS-CoV-2, including the delta and omicron variants. Viruses. 2022;14(7):1369. doi: 10.3390/v14071369 35891350 PMC9318744

[28] Sasaki M, Kishimoto M, Itakura Y, Tabata K, Intaruck K, Uemura K, et al. Air-liquid interphase culture confers SARS-CoV-2 susceptibility to A549 alveolar epithelial cells. Biochem Biophys Res Commun. 2021;577:146–51. doi: 10.1016/j.bbrc.2021.09.015 34517212 PMC8423671

[29] Sherwood AV, Rivera-Rangel LR, Ryberg LA, Larsen HS, Anker KM, Costa R, et al. Hepatitis C virus RNA is 5’-capped with flavin adenine dinucleotide. Nature. 2023;619(7971):811–8. doi: 10.1038/s41586-023-06301-3 37407817 PMC7616780

[30] Yang W, Qiu C, Biswas N, Jin J, Watkins SC, Montelaro RC, et al. Correlation of the tight junction-like distribution of Claudin-1 to the cellular tropism of hepatitis C virus. J Biol Chem. 2008;283(13):8643–53. doi: 10.1074/jbc.M709824200 18211898 PMC2417170

[31] Chung H, Noh JY, Koo B-S, Hong JJ, Kim HK. SARS-CoV-2 mutations acquired during serial passage in human cell lines are consistent with several of those found in recent natural SARS-CoV-2 variants. Comput Struct Biotechnol J. 2022;20:1925–34. doi: 10.1016/j.csbj.2022.04.022 35474907 PMC9021118

[32] Yan K, Dumenil T, Tang B, Le TT, Bishop CR, Suhrbier A, et al. Evolution of ACE2-independent SARS-CoV-2 infection and mouse adaption after passage in cells expressing human and mouse ACE2. Virus Evol. 2022;8(2):veac063. doi: 10.1093/ve/veac063 35919871 PMC9338707

[33] Yan K, Dumenil T, Stewart R, Bishop CR, Tang B, Nguyen W, et al. TMEM106B-mediated SARS-CoV-2 infection allows for robust ACE2-independent infection in vitro but not in vivo. Cell Rep. 2024;43(11). 10.1016/j.celrep.2024.114921 39480813

[34] Clausen TM, Sandoval DR, Spliid CB, Pihl J, Perrett HR, Painter CD, et al. SARS-CoV-2 infection depends on cellular heparan sulfate and ACE2. Cell. 2020;183(4):1043-1057.e15. doi: 10.1016/j.cell.2020.09.033 32970989 PMC7489987

[35] Pham LV, Underwood AP, Binderup A, Fahnøe U, Fernandez-Antunez C, Lopez-Mendez B, et al. Neutralisation resistance of SARS-CoV-2 spike-variants is primarily mediated by synergistic receptor binding domain substitutions. Emerg Microbes Infect. 2024;13(1):2412643. doi: 10.1080/22221751.2024.2412643 39392057 PMC11562025

[36] Sagar S, Rathinavel AK, Lutz WE, Struble LR, Khurana S, Schnaubelt AT, et al. Bromelain inhibits SARS-CoV-2 infection via targeting ACE-2, TMPRSS2, and spike protein. Clin Transl Med. 2021;11(2):e281. doi: 10.1002/ctm2.281 33635001 PMC7811777

[37] Lamers MM, Mykytyn AZ, Breugem TI, Wang Y, Wu DC, Riesebosch S, et al. Human airway cells prevent SARS-CoV-2 multibasic cleavage site cell culture adaptation. Elife. 2021;10:e66815. doi: 10.7554/eLife.66815 33835028 PMC8131099

[38] Chaudhry MZ, Eschke K, Hoffmann M, Grashoff M, Abassi L, Kim Y, et al. Rapid SARS-CoV-2 adaptation to available cellular proteases. J Virol. 2022;96(5):e0218621. doi: 10.1128/jvi.02186-21 35019723 PMC8906416

[39] Hossain MS, Tonmoy MIQ, Fariha A, Islam MS, Roy AS, Islam MN, et al. Prediction of the effects of variants and differential expression of key host genes ACE2, TMPRSS2, and FURIN in SARS-CoV-2 pathogenesis: an in silico approach. Bioinform Biol Insights. 2021;15:11779322211054684. doi: 10.1177/11779322211054684 34720581 PMC8554545

[40] Ma Y, Li P, Hu Y, Qiu T, Wang L, Lu H, et al. Spike substitution T813S increases Sarbecovirus fusogenicity by enhancing the usage of TMPRSS2. PLoS Pathog. 2023;19(5):e1011123. doi: 10.1371/journal.ppat.1011123 37196033 PMC10228797

[41] Pastorio C, Zech F, Noettger S, Jung C, Jacob T, Sanderson T, et al. Determinants of Spike infectivity, processing, and neutralization in SARS-CoV-2 Omicron subvariants BA.1 and BA.2. Cell Host Microbe. 2022;30(9):1255-1268.e5. doi: 10.1016/j.chom.2022.07.006 35931073 PMC9289044

[42] Parsons RJ, Acharya P. Evolution of the SARS-CoV-2 Omicron spike. Cell Rep. 2023;42(12):113444. doi: 10.1016/j.celrep.2023.113444 37979169 PMC10782855

