# Supplementary figures and images for: Spike substitutions E484D, P812R and Q954H mediate ACE2-independent entry of SARS-CoV-2 across different cell lines
Source: PLoS One. 2025 Aug 1;20(8):e0326419. doi: 10.1371/journal.pone.0326419 (PMC12316203; doi:10.1371/journal.pone.0326419)

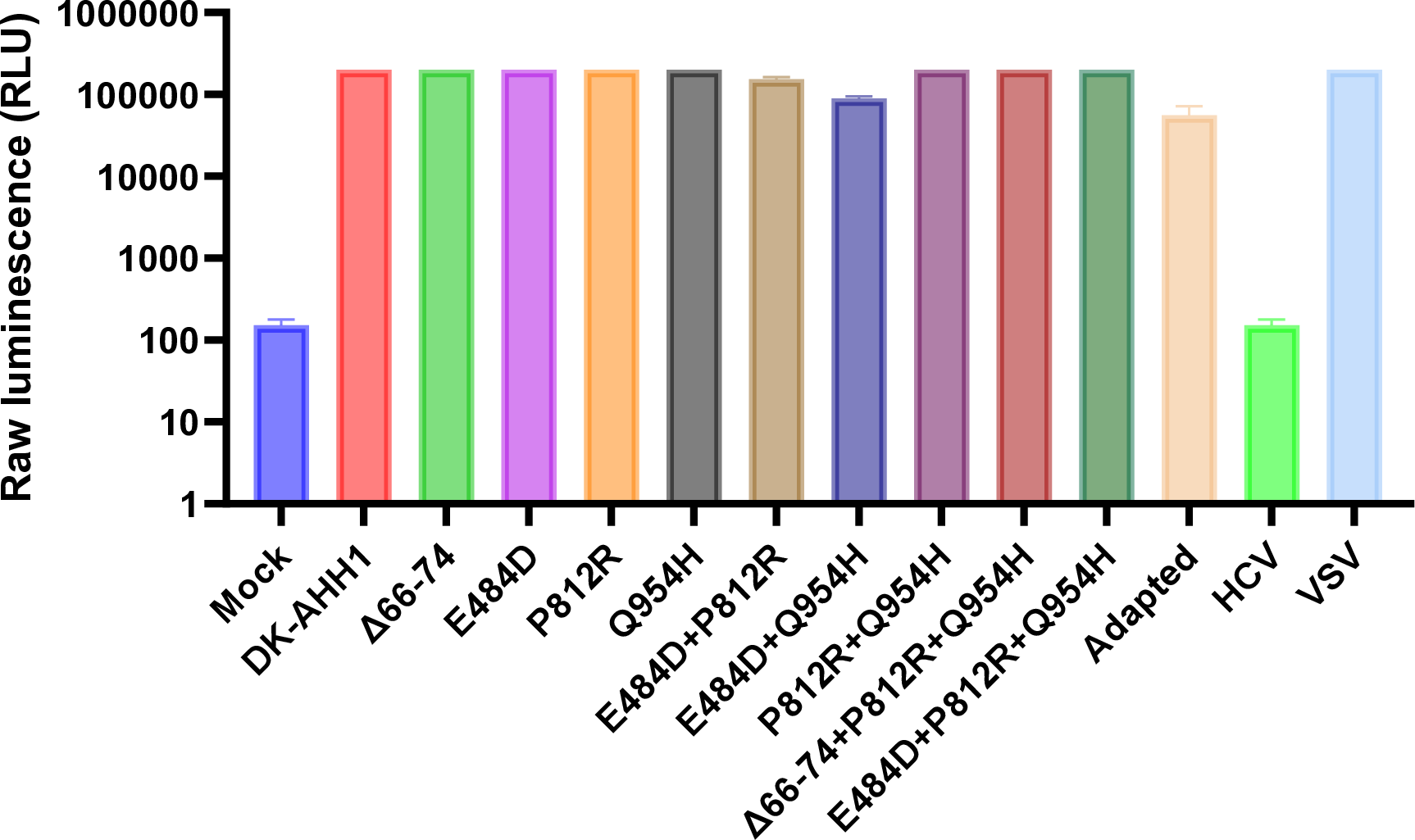

Supplement: S1 Fig — (TIF) [file pone.0326419.s001.tif]

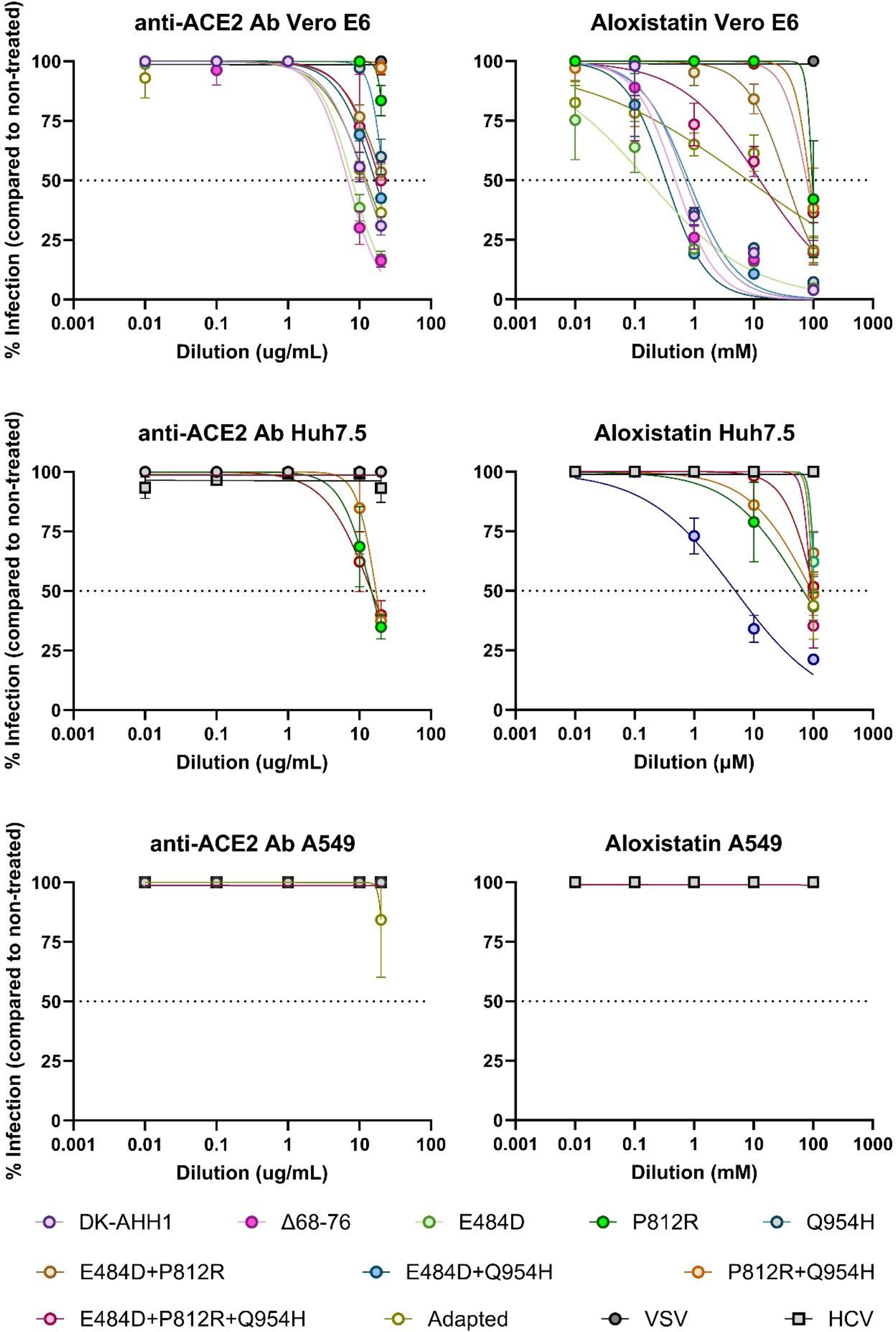

Supplement: S2 Fig — The error bars represent the standard deviation from the calculated percentage infection of the four pseudoparticle replicates. All calculated 50% inhibitory values can be found in S13 Table. (TIF) [file pone.0326419.s002.tif]

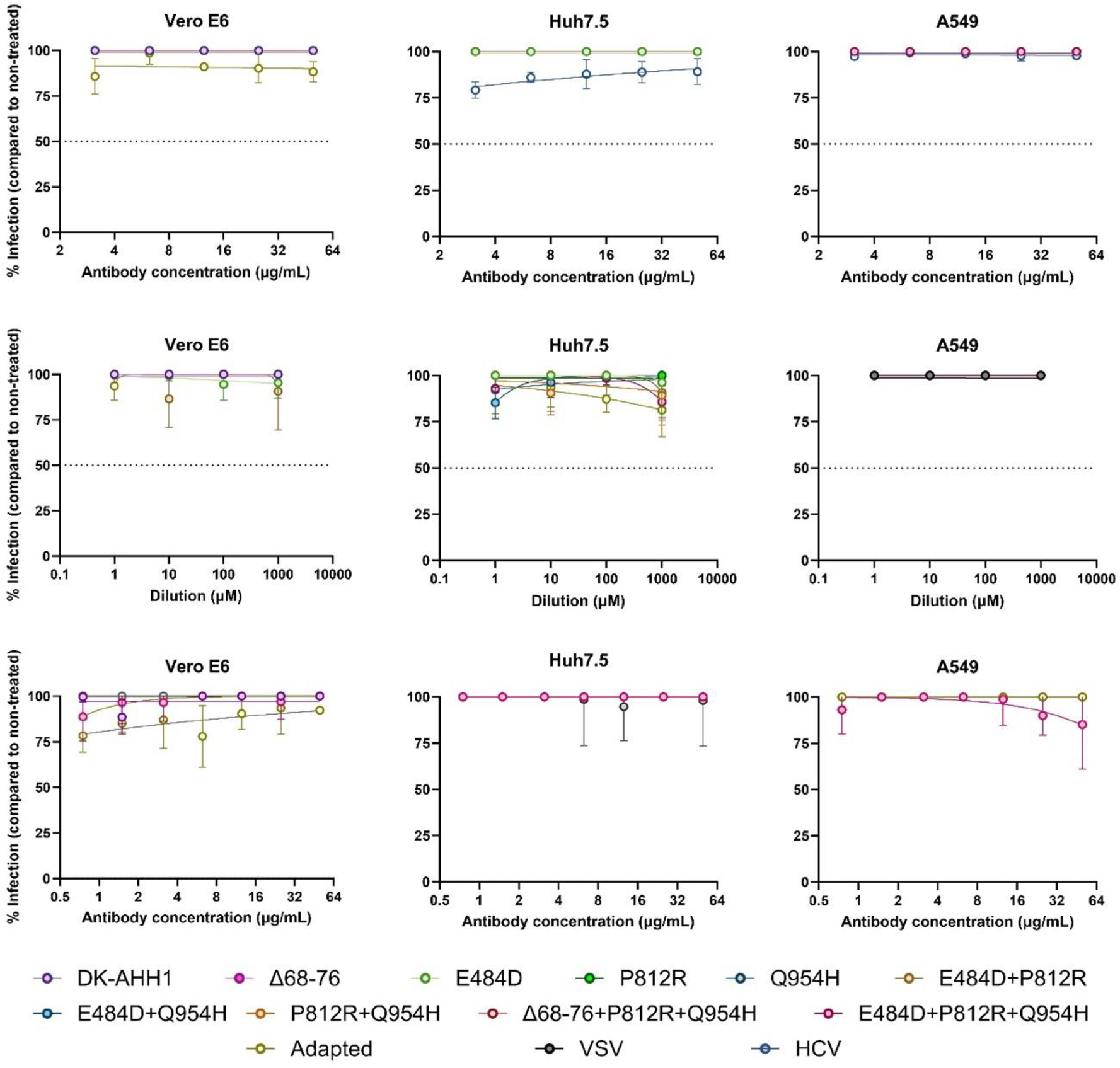

Supplement: S3 Fig — The error bars represent the standard deviation from the calculated percentage infection of the four pseudoparticle replicates. (TIF) [file pone.0326419.s003.tif]
