# Supplementary material for: Spike substitutions E484D, P812R and Q954H mediate ACE2-independent entry of SARS-CoV-2 across different cell lines
Source: PLoS One. 2025 Aug 1;20(8):e0326419. doi: 10.1371/journal.pone.0326419 (PMC12316203; doi:10.1371/journal.pone.0326419)
Supplement: S1 Table — (DOCX) [file pone.0326419.s004.docx]

**Supplementary Table 1. Infectivity of each pseudoparticle measured as signal/noise in relative luminescence units (RLUs) plotted in Figure 1.**

|  | Vero E6 | | Huh7.5 | | A549 | | A549-ACE2 | |
| --- | --- | --- | --- | --- | --- | --- | --- | --- |
|  | **Mean** | **SD** | **Mean** | **SD** | **Mean** | **SD** | **Mean** | **SD** |
| DK-AHH1 | 176 | 0 | 4 | 3 | 1 | 0 | 1230 | 21 |
| Δ68-76 | 176 | 0 | 2 | 1 | 1 | 0 | 158 | 8 |
| E484D | 176 | 0 | 17 | 1 | 1 | 0 | 428 | 121 |
| P812R | 146 | 0 | 45 | 9 | 2 | 1 | 1242 | 0 |
| Q954H | 146 | 0 | 7 | 4 | 1 | 0 | 1242 | 0 |
| E484D+P812R | 77 | 21 | 118 | 3 | 8 | 1 | 631 | 0 |
| E484D+Q954H | 45 | 14 | 92 | 15 | 2 | 1 | 182 | 25 |
| P812R+Q954H | 146 | 0 | 76 | 13 | 3 | 1 | 1077 | 88 |
| Δ68-76+P812R+Q954H | 169 | 0 | 34 | 8 | 3 | 0 | 1135 | 42 |
| E484D+P812R+Q954H | 169 | 0 | 60 | 1 | 135 | 16 | 996 | 105 |
| Adapted | 165 | 4 | 36 | 6 | 85 | 13 | 274 | 32 |
| HCV | 1 | 0 | 293 | 18 | 224 | 31 | 191 | 27 |
| VSV | 338 | 0 | 257 | 37 | 221 | 0 | 242 | 0 |
