# Supplementary material for: Spike substitutions E484D, P812R and Q954H mediate ACE2-independent entry of SARS-CoV-2 across different cell lines
Source: PLoS One. 2025 Aug 1;20(8):e0326419. doi: 10.1371/journal.pone.0326419 (PMC12316203; doi:10.1371/journal.pone.0326419)
Supplement: S2 Table — (DOCX) [file pone.0326419.s005.docx]

**Supplementary Table 2. The percentage (%) infection values (compared to the non-treated control) plotted in Figure 2A (Vero E6 cells).**

|  | siRNA | | ACE2 blocking (20µg/mL) | | EC_50_ Aloxistatin | |
| --- | --- | --- | --- | --- | --- | --- |
|  | **Mean** | **SD** | **Mean** | **SD** | **Mean** | **SD** |
| DK-AHH1 | 43 | 17 | 31 | 4 | 20 | 2 |
| Δ68-76 | 38 | 8 | 16 | 3 | 17 | 2 |
| E484D | 26 | 4 | 17 | 3 | 16 | 1 |
| P812R | 92 | 6 | 84 | 6 | 100 | 0 |
| Q954H | 43 | 6 | 60 | 7 | 22 | 2 |
| E484D+P812R | 52 | 6 | 43 | 1 | 84 | 6 |
| E484D+Q954H | 61 | 4 | 54 | 3 | 11 | 1 |
| P812R+Q954H | 91 | 5 | 97 | 3 | 100 | 0 |
| Δ68-76+P812R+Q954H | 75 | 11 | 97 | 3 | 99 | 1 |
| E484D+P812R+Q954H | 25 | 8 | 50 | 8 | 58 | 6 |
| Adapted | 54 | 8 | 36 | 4 | 61 | 8 |
| VSV | 100 | 0 | 125 | 50 | 100 | 0 |
