# Supplementary material for: Spike substitutions E484D, P812R and Q954H mediate ACE2-independent entry of SARS-CoV-2 across different cell lines
Source: PLoS One. 2025 Aug 1;20(8):e0326419. doi: 10.1371/journal.pone.0326419 (PMC12316203; doi:10.1371/journal.pone.0326419)
Supplement: S3 Table — (DOCX) [file pone.0326419.s006.docx]

**Supplementary Table 3. The percentage (%) infection values (compared to the non-treated control) plotted in Figure 2B (Huh7.5 cells).**

|  | siRNA | | ACE2 blocking (20µg/mL) | | EC_50_ Aloxistatin | |
| --- | --- | --- | --- | --- | --- | --- |
|  | **Mean** | **SD** | **Mean** | **SD** | **Mean** | **SD** |
| E484D | 71 | 6 | 100 | 0 | 62 | 12 |
| P812R | 39 | 9 | 35 | 5 | 43 | 6 |
| E484D+P812R | 64 | 9 | 87 | 8 | 66 | 9 |
| E484D+Q954H | 69 | 6 | 100 | 0 | 28 | 6 |
| P812R+Q954H | 38 | 9 | 37 | 3 | 21 | 2 |
| Δ68-76+P812R+Q954H | 31 | 5 | 40 | 6 | 61 | 13 |
| E484D+P812R+Q954H | 85 | 6 | 100 | 0 | 100 | 0 |
| Adapted | 75 | 7 | 100 | 0 | 100 | 0 |
| HCV | 100 | 0 | 100 | 0 | 100 | 0 |
| VSV | 100 | 0 | 100 | 0 | 100 | 0 |
