# Supplementary material for: Spike substitutions E484D, P812R and Q954H mediate ACE2-independent entry of SARS-CoV-2 across different cell lines
Source: PLoS One. 2025 Aug 1;20(8):e0326419. doi: 10.1371/journal.pone.0326419 (PMC12316203; doi:10.1371/journal.pone.0326419)
Supplement: S4 Table — (DOCX) [file pone.0326419.s007.docx]

**Supplementary Table 4. The percentage (%) infection values (compared to the non-treated control) plotted in Figure 2C (A549 cells).**

|  | siRNA | | ACE2 blocking (20µg/mL) | | EC_50_ Aloxistatin | |
| --- | --- | --- | --- | --- | --- | --- |
|  | **Mean** | **SD** | **Mean** | **SD** | **Mean** | **SD** |
| E484D+P812R+Q954H | 65 | 25 | 100 | 0 | 100 | 0 |
| Adapted | 72 | 11 | 84 | 24 | 100 | 0 |
| HCV | 77 | 33 | 100 | 0 | 100 | 0 |
| VSV | 100 | 0 | 100 | 0 | 100 | 0 |
