# Supplementary material for: Spike substitutions E484D, P812R and Q954H mediate ACE2-independent entry of SARS-CoV-2 across different cell lines
Source: PLoS One. 2025 Aug 1;20(8):e0326419. doi: 10.1371/journal.pone.0326419 (PMC12316203; doi:10.1371/journal.pone.0326419)
Supplement: S5 Table — (DOCX) [file pone.0326419.s008.docx]

**Supplementary Table 5. The percentage (%) infection values (compared to the non-treated control) plotted in Figure 3A (Vero E6 cells).**

|  | Aloxistatin (25μM) | | Aloxistatin (25μM) + Camostat (500μM) | |
| --- | --- | --- | --- | --- |
|  | **Mean** | **SD** | **Mean** | **SD** |
| DK-AHH1 | 5 | 0 | 7 | 1 |
| Δ68-76 | 9 | 0 | 12 | 3 |
| E484D | 7 | 0 | 6 | 1 |
| P812R | 87 | 0 | 78 | 13 |
| Q954H | 20 | 0 | 21 | 6 |
| E484D+P812R | 33 | 0 | 52 | 15 |
| E484D+Q954H | 7 | 0 | 9 | 2 |
| P812R+Q954H | 100 | 0 | 93 | 9 |
| Δ68-76+P812R+Q954H | 100 | 0 | 100 | 0 |
| E484D+P812R+Q954H | 40 | 0 | 44 | 5 |
| Adapted | 43 | 0 | 34 | 9 |
| HCV | 0 | 0 | 0 | 0 |
| VSV | 100 | 0 | 100 | 0 |
