# Supplementary material for: Spike substitutions E484D, P812R and Q954H mediate ACE2-independent entry of SARS-CoV-2 across different cell lines
Source: PLoS One. 2025 Aug 1;20(8):e0326419. doi: 10.1371/journal.pone.0326419 (PMC12316203; doi:10.1371/journal.pone.0326419)
Supplement: S6 Table — (DOCX) [file pone.0326419.s009.docx]

**Supplementary Table 6. The percentage (%) infection values (compared to the non-treated control) plotted in Figure 3B (Huh7.5 cells).**

|  | Aloxistatin (25μM) | | Aloxistatin (25μM) + Camostat (500μM) | |
| --- | --- | --- | --- | --- |
|  | **Mean** | **SD** | **Mean** | **SD** |
| E484D | 94 | 0 | 47 | 8 |
| P812R | 73 | 0 | 78 | 13 |
| E484D+P812R | 94 | 0 | 79 | 4 |
| E484D+Q954H | 32 | 0 | 21 | 4 |
| P812R+Q954H | 80 | 0 | 72 | 17 |
| Δ68-76+P812R+Q954H | 90 | 0 | 64 | 22 |
| E484D+P812R+Q954H | 91 | 0 | 84 | 7 |
| Adapted | 91 | 0 | 94 | 15 |
| HCV | 100 | 0 | 97 | 8 |
| VSV | 100 | 0 | 100 | 0 |
