# Supplementary material for: Spike substitutions E484D, P812R and Q954H mediate ACE2-independent entry of SARS-CoV-2 across different cell lines
Source: PLoS One. 2025 Aug 1;20(8):e0326419. doi: 10.1371/journal.pone.0326419 (PMC12316203; doi:10.1371/journal.pone.0326419)
Supplement: S8 Table — (DOCX) [file pone.0326419.s011.docx]

**Supplementary Table 8. The percentage (%) neutralization values for neutralization of DK-AHH1 in Vero E6 cells (Figure 4A).**

|  | Non-H-51 | | Non-H-10 | | Non-H-17 | | Non-H-57 | | Non-H-12 | | Non-H-05 | |
| --- | --- | --- | --- | --- | --- | --- | --- | --- | --- | --- | --- | --- |
| Log dilution | **Mean** | **SD** | **Mean** | **SD** | **Mean** | **SD** | **Mean** | **SD** | **Mean** | **SD** | **Mean** | **SD** |
| 1,30 | 94 | 3 | 99 | 0 | 92 | 2 | 48 | 4 | 64 | 4 | 40 | 5 |
| 1,60 | 71 | 5 | 92 | 1 | 75 | 2 | 17 | 4 | 21 | 10 | 19 | 1 |
| 1,90 | 51 | 1 | 65 | 9 | 46 | 3 | 0 | 0 | 0 | 0 | 0 | 0 |
| 2,20 | 35 | 8 | 35 | 12 | 19 | 8 | 0 | 0 | 0 | 0 | 0 | 0 |
| 2,51 | 29 | 2 | 20 | 2 | 8 | 12 | 0 | 0 | 0 | 0 | 0 | 0 |
| 2,81 | 0 | 0 | 0 | 0 | 0 | 0 | 0 | 0 | 0 | 0 | 0 | 0 |
| 3,11 | 0 | 0 | 0 | 0 | 0 | 0 | 0 | 0 | 0 | 0 | 0 | 0 |
