# Supplementary material for: Spike substitutions E484D, P812R and Q954H mediate ACE2-independent entry of SARS-CoV-2 across different cell lines
Source: PLoS One. 2025 Aug 1;20(8):e0326419. doi: 10.1371/journal.pone.0326419 (PMC12316203; doi:10.1371/journal.pone.0326419)
Supplement: S9 Table — (DOCX) [file pone.0326419.s012.docx]

**Supplementary Table 9. The percentage (%) neutralization values for neutralization of the adapted variant in Vero E6 cells (Figure 4B).**

|  | Non-H-51 | | Non-H-10 | | Non-H-17 | | Non-H-57 | | Non-H-12 | | Non-H-05 | |
| --- | --- | --- | --- | --- | --- | --- | --- | --- | --- | --- | --- | --- |
| Log dilution | **Mean** | **SD** | **Mean** | **SD** | **Mean** | **SD** | **Mean** | **SD** | **Mean** | **SD** | **Mean** | **SD** |
| 1,30 | 70 | 11 | 76 | 6 | 66 | 6 | 71 | 8 | 76 | 14 | 81 | 13 |
| 1,60 | 59 | 8 | 60 | 16 | 54 | 4 | 60 | 10 | 59 | 8 | 74 | 8 |
| 1,90 | 34 | 5 | 40 | 6 | 37 | 9 | 36 | 11 | 29 | 18 | 58 | 7 |
| 2,20 | 9 | 8 | 29 | 11 | 25 | 13 | 8 | 7 | 0 | 0 | 29 | 3 |
| 2,51 | 7 | 8 | 31 | 12 | 31 | 15 | 0 | 0 | 17 | 25 | 28 | 6 |
| 2,81 | 1 | 7 | 36 | 13 | 14 | 20 | 0 | 0 | 16 | 19 | 2 | 28 |
| 3,11 | 0 | 0 | 24 | 19 | 19 | 26 | 0 | 0 | 0 | 0 | -6 | 30 |
