# Supplementary material for: Spike substitutions E484D, P812R and Q954H mediate ACE2-independent entry of SARS-CoV-2 across different cell lines
Source: PLoS One. 2025 Aug 1;20(8):e0326419. doi: 10.1371/journal.pone.0326419 (PMC12316203; doi:10.1371/journal.pone.0326419)
Supplement: S10 Table — (DOCX) [file pone.0326419.s013.docx]

**Supplementary Table 10. The percentage (%) neutralization values for neutralization of the adapted variant in Huh7.5 cells (Figure 4B).**

|  | Non-H-51 | | Non-H-10 | | Non-H-17 | | Non-H-57 | | Non-H-12 | | Non-H-05 | |
| --- | --- | --- | --- | --- | --- | --- | --- | --- | --- | --- | --- | --- |
| Log dilution | **Mean** | **SD** | **Mean** | **SD** | **Mean** | **SD** | **Mean** | **SD** | **Mean** | **SD** | **Mean** | **SD** |
| 1,30 | 96 | 1 | 97 | 3 | 95 | 2 | 75 | 14 | 91 | 4 | 97 | 3 |
| 1,60 | 88 | 6 | 80 | 10 | 82 | 6 | 37 | 11 | 71 | 9 | 85 | 4 |
| 1,90 | 47 | 4 | 48 | 15 | 59 | 13 | 10 | 3 | 41 | 18 | 58 | 18 |
| 2,20 | 1 | 3 | 28 | 15 | 37 | 5 | 0 | 0 | 26 | 10 | 56 | 13 |
| 2,51 | 0 | 0 | 28 | 9 | 28 | 11 | 0 | 0 | 26 | 10 | 34 | 10 |
| 2,81 | 0 | 0 | 26 | 11 | 0 | 0 | 0 | 0 | 22 | 4 | 14 | 7 |
| 3,11 | 0 | 0 | 14 | 13 | 0 | 0 | 0 | 0 | 11 | 14 | 5 | 3 |
