# Supplementary material for: Spike substitutions E484D, P812R and Q954H mediate ACE2-independent entry of SARS-CoV-2 across different cell lines
Source: PLoS One. 2025 Aug 1;20(8):e0326419. doi: 10.1371/journal.pone.0326419 (PMC12316203; doi:10.1371/journal.pone.0326419)
Supplement: S11 Table — (DOCX) [file pone.0326419.s014.docx]

**Supplementary Table 11. The percentage neutralization values for neutralization of the adapted variant in A549 cells (Figure 4B).**

|  | Non-H-51 | | Non-H-10 | | Non-H-17 | | Non-H-57 | | Non-H-12 | | Non-H-05 | |
| --- | --- | --- | --- | --- | --- | --- | --- | --- | --- | --- | --- | --- |
| Log dilution | **Mean** | **SD** | **Mean** | **SD** | **Mean** | **SD** | **Mean** | **SD** | **Mean** | **SD** | **Mean** | **SD** |
| 1,30 | 94 | 6 | 106 | 1 | 99 | 6 | 99 | 3 | 98 | 7 | 92 | 17 |
| 1,60 | 74 | 20 | 89 | 18 | 56 | 25 | 68 | 7 | 85 | 12 | 96 | 14 |
| 1,90 | 22 | 12 | 54 | 15 | 19 | 64 | 29 | 13 | 66 | 26 | 87 | 16 |
| 2,20 | 14 | 14 | 0 | 0 | 26 | 56 | 12 | 26 | 27 | 26 | 72 | 12 |
| 2,51 | 0 | 0 | 0 | 0 | 33 | 57 | 0 | 0 | 0 | 0 | 21 | 6 |
| 2,81 | 0 | 0 | 0 | 0 | 0 | 0 | 0 | 0 | 0 | 0 | 14 | 40 |
| 3,11 | 0 | 0 | 0 | 0 | 0 | 0 | 0 | 0 | 0 | 0 | 0 | 0 |
