# Supplementary material for: Spike substitutions E484D, P812R and Q954H mediate ACE2-independent entry of SARS-CoV-2 across different cell lines
Source: PLoS One. 2025 Aug 1;20(8):e0326419. doi: 10.1371/journal.pone.0326419 (PMC12316203; doi:10.1371/journal.pone.0326419)
Supplement: S12 Table — (DOCX) [file pone.0326419.s015.docx]

**Supplementary Table 12. ID_50_ values for the DK-AHH1 and adapted pseudoparticle across cell lines.**

| Subject ID | Vero E6 | | Huh7.5 | A549 |
| --- | --- | --- | --- | --- |
|  | DK-AHH1 | Adapted | Adapted | Adapted |
| Non-H-05 | 82 | 45 | 76 | 56 |
| Non-H-10 | 136 | 74 | 109 | 81 |
| Non-H-12 | 77 | 46 | 118 | 56 |
| Non-H-17 | 19 | 46 | 32 | 57 |
| Non-H-51 | 24 | 49 | 85 | 102 |
| Non-H-57 | 17 | 92 | 158 | 117 |
