# Supplementary material for: Spike substitutions E484D, P812R and Q954H mediate ACE2-independent entry of SARS-CoV-2 across different cell lines
Source: PLoS One. 2025 Aug 1;20(8):e0326419. doi: 10.1371/journal.pone.0326419 (PMC12316203; doi:10.1371/journal.pone.0326419)
Supplement: S13 Table — (DOCX) [file pone.0326419.s016.docx]

**Supplementary Table 13. IC_50_ (anti-ACE2 antibody) and EC_50_ (aloxistatin) values for the panel of pseudoparticle across cell lines.**

| Spike variant | Vero E6 | | Huh7.5 | | A549 | |
| --- | --- | --- | --- | --- | --- | --- |
|  | **Anti-ACE2** | **Aloxistatin** | **Anti-ACE2** | **Aloxistatin** | **Anti-ACE2** | **Aloxistatin** |
| DK-AHH1 | 12 | 1 | N/A | N/A | N/A | N/A |
| Δ68-76 | 7 | 0 | N/A | N/A | N/A | N/A |
| E484D | 8 | 0 | >20 | 105 | N/A | N/A |
| P812R | 24 | 96 | 15 | 68 | N/A | N/A |
| Q954H | 22 | 1 | N/A | N/A | N/A | N/A |
| P812R+Q954H | 31 | 87 | 17 | 94 | N/A | N/A |
| Δ68-76+P812R+Q954H | 32 | 78 | 15 | 104 | N/A | N/A |
| E484D+P812R+Q954H | 19 | 13 | >20 | 91 | >20 | >100 |
| Adapted | 12 | 8 | >20 | 97 | 23 | >100 |
| E484D+P812R | 22 | 35 | >20 | 107 | N/A | N/A |
| E484D+Q954H | 17 | 0 | >20 | 5 | N/A | N/A |
| VSV | >20 | >100 | >20 | >100 | >20 | >100 |
| HCV | N/A | N/A | >20 | >100 | >20 | >100 |

N/A=not applicable.
